# Suppression of 14-3-3γ-mediated surface expression of ANO1 inhibits cancer progression of glioblastoma cells

**DOI:** 10.1038/srep26413

**Published:** 2016-05-23

**Authors:** Young-Sun Lee, Jae Kwang Lee, Yeonju Bae, Bok-Soon Lee, Eunju Kim, Chang-Hoon Cho, Kanghyun Ryoo, Jiyun Yoo, Chul-Ho Kim, Gwan-Su Yi, Seok-Geun Lee, C. Justin Lee, Sang Soo Kang, Eun Mi Hwang, Jae-Yong Park

**Affiliations:** 1 School of Biosystem and Biomedical Science, College of Health Science, Korea University, Seoul 02841, Republic of Korea; 2 Department of Anatomy and Convergence Medical Science, Institute of Health Sciences, School of Medicine, Gyeongsang National University, Jinju 52727, Republic of Korea; 3 Center for Functional Connectomics, Korea Institute of Science and Technology (KIST), Seoul 02792, Republic of Korea; 4 Neuroscience Program, University of Science and Technology (UST), Daejeon 34113, Republic of Korea; 5 Department of Otolaryngology, Ajou University School of Medicine, Suwon 16499, Republic of Korea; 6 Division of Applied Life Science (BK21 plus), Research Institute of Life Sciences, Gyeongsang National University, Jinju 52828, Korea; 7 Department of Bio and Brain Engineering, KAIST, Daejeon 34141, Republic of Korea; 8 Department of Science in Korean Medicine, College of Korean Medicine, KHU-KIST department of Convergging Science and Technology, Kyung Hee University, Seoul 02447, Republic of Korea

## Abstract

Anoctamin-1 (ANO1) acts as a Ca^2+^-activated Cl^−^ channel in various normal tissues, and its expression is increased in several different types of cancer. Therefore, understanding the regulation of ANO1 surface expression is important for determining its physiological and pathophysiological functions. However, the trafficking mechanism of ANO1 remains elusive. Here, we report that *segment a* (N-terminal 116 amino acids) of ANO1 is crucial for its surface expression, and we identified 14-3-3γ as a binding partner for anterograde trafficking using yeast two-hybrid screening. The surface expression of ANO1 was enhanced by 14-3-3γ, and the Thr9 residue of ANO1 was critical for its interaction with 14-3-3γ. Gene silencing of 14-3-3γ and/or ANO1 demonstrated that suppression of ANO1 surface expression inhibited migration and invasion of glioblastoma cells. These findings provide novel therapeutic implications for glioblastomas, which are associated with poor prognosis.

Ca^2+^-activated Cl^−^ channels (CaCCs), which are activated by intracellular Ca^2+^, have crucial roles in physiological processes such as epithelial secretion, smooth muscle excitability, olfactory perception, cardiac excitability, and nociception^1^,^2^. In 2008, three independent groups identified ANO1 (anoctamin-1, also known as TMEM16A) as a CaCC^3^,^4^,^5^. ANO1 contains eight transmembrane domains (TMs), a pore-loop between TM5 and TM6, and cytosolic N- and C-termini which were predicted by hydropathy analysis^3^. Interestingly, an X-ray crystal structure revealed that a TMEM16 in fungus, *Nectria haematococca* (nhTMEM16) has ten TMs^6^.

ANO1 is expressed in many different tissues with demonstrated CaCC activity, where it participates in various cellular processes. ANO1 plays important roles in fluid secretion and cell volume regulation upon osmotic stress in epithelial cells of the salivary gland, trachea, pancreas, gut, and mammary gland^7^,^8^,^9^,^10^,^11^,^12^. ANO1 also acts as a pacemaker for spontaneous activity of the interstitial cells of Cajal^13^ and a heat sensor in dorsal root ganglion neurons^14^. ANO1 is involved in inflammatory and nerve injury-induced hypersensitivity^15^ and in testosterone-induced prostate hyperplasis^16^. In addition, ANO1 is overexpressed in various cancer cells of the breast, pancreas, urinary bladder, esophagus, and prostate, as well as ovarian tumors, parathyroid tumors, head and neck squamous cell carcinoma (HNSCC), pancreatic tumors, and glioblastoma^11^,^17^,^18^,^19^,^20^,^21^,^22^.

Based on reports of ANO1 overexpression in various types of cancers, it is likely that ANO1 is important for cancer development and metastasis. Recent studies show that ANO1 in HNSCC contributes to tumorigenesis and invasion and enhances epidermal growth factor (EGF) receptor (EGFR) signaling by interacting with EGFR^23^. ANO1 also promotes cancer progression by stimulating the cell proliferation signaling pathway involving EGFR and calmodulin-dependent protein kinase II (CAMKII) in breast cancer cells^17^. In addition, inhibition of ANO1 suppresses proliferation, migration, and invasion of human lung cancer and glioblastoma^22^,^24^.

Our previous study demonstrated that activation of various receptor tyrosine kinases and G protein-coupled receptors increase intracellular Ca^2+^ concentration through the phosphoinositide pathway in glioblastoma cells, and that caffeine inhibits increases in intracellular Ca^2+^ and in turn suppresses migration and invasion of glioblastoma cells^25^. As ANO1 is activated by intracellular Ca^2+^, it is plausible that receptor-mediated increases in intracellular Ca^2+^ can activate ANO1 channels, which might promote ANO1-mediated cancer progression of glioblastoma cells. Because most ion channels act at the plasma membrane, clarifying the trafficking mechanisms of ANO1 channels at the plasma membrane is important for developing potent therapeutic approaches for glioblastomas. In addition, understanding the molecular mechanisms of ANO1 trafficking will help expand our knowledge of the physiological roles of ANO1 in various tissues and other ANO1-related diseases.

Different isoforms of the ANO1 channel are generated by alternative splicing^5^,^26^,^27^. The alternative sequences coding protein segments are *segment a* (116 residues), *b* (22 residues), *c* (4 residues), and *d* (26 residues), which generate several ANO1 isoforms such as (*a*), (*ac*), (*abc*), (*acd*), (*abcd*), and (*0*). *Segments a* and *b* are localized in the N-terminus, whereas *segments c* and *d* are in the first intracellular loop. *Segments b* and *c* are involved in Ca^2+^ sensitivity and voltage dependence of ANO1, respectively^27^. In addition, *segment a* has been shown to be important for the channel activity of ANO1^28^.

In the present study, we demonstrated that *segment a* is a critical domain for the surface expression of ANO1. We identified 14-3-3γ as a binding partner for *segment a* using yeast two-hybrid (Y2H) screening and found that the surface expression of ANO1 is enhanced by interactions with 14-3-3γ. Suppression of ANO1 surface expression using 14-3-3γ-specific short hairpin RNA (shRNA) reduced ANO1-mediated channel activity in glioblastoma such as U251, T98G and U138 cell lines. In addition, gene silencing of 14-3-3γ and/or ANO1 inhibited migration and invasion of these glioblastoma cell lines. These findings provide novel insights for understanding tumorous characteristics of ANO1-related cancers.

## Results

### *Segment a* is essential for the surface expression of ANO1

**Figure 1:**
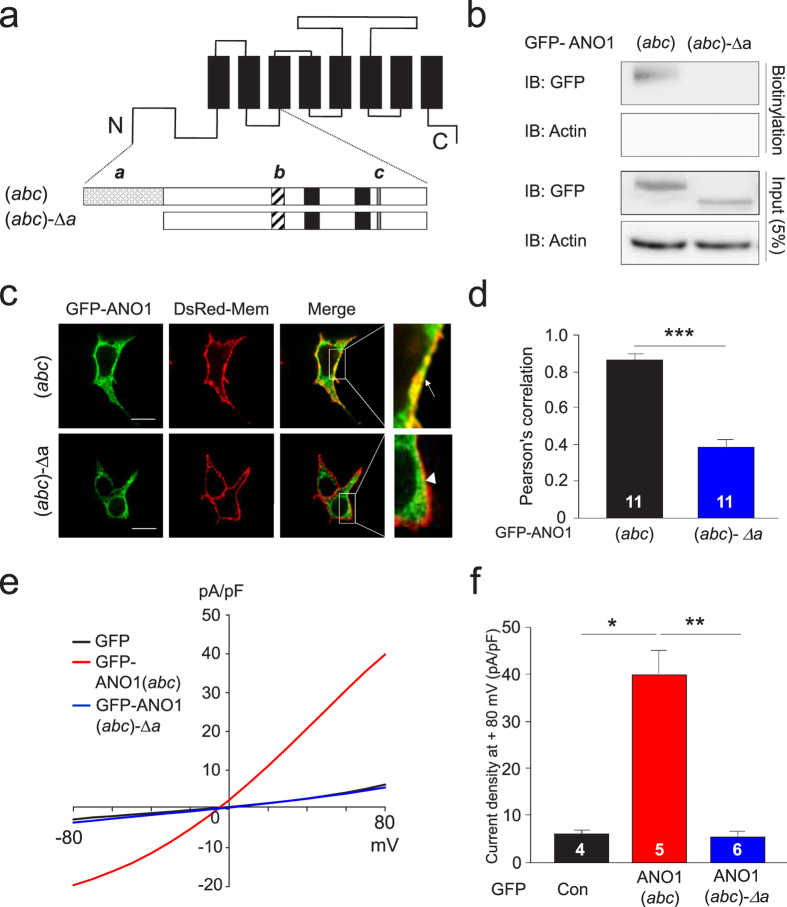
*Segment a* of ANO1 is critical for the expression of ANO1 at the plasma membrane. (**a**) Schematic representation of ANO1(*abc*) isoform and its deletion mutant. Black boxes: transmembrane domains; hatched box: *segment a*; diagonal striped box: *segment b*, gray box: *segment c*. (**b**) Cell surface biotinylation showed that *segment a* is critical for the surface expression of ANO1 in GFP-ANO1(*abc*)*-* or GFP-ANO1(*abc*)*-Δa*-transfected HEK293T cells. Western blot images were cropped for clarification. Full blot images were included in supplementary information. (**c**) Co-localization of GFP-ANO1(*abc*) or GFP-ANO1(*abc*)*-Δa* with DsRed-Mem in HEK293T cells. Scale bars, 10 μm. The presence and absence of co-localized GFP signals and membrane markers are shown as an arrow and arrowhead, respectively, in the enlarged images. (**d**) Pearson’s correlation coefficients denoting covariance of fluorescence signals were calculated using Nikon software as in (**c**). Data are expressed as mean ± SEM; ***P < 0.001. (**e**) Representative traces of whole-cell recordings of COS7 cells transfected with GFP-Con, GFP-ANO1(*abc*), or GFP-ANO1(*abc*)*-Δa*. (**f**) Summary bar graph showing normalized current density as in (**e**) at +80 mV. Data are expressed as mean ± SEM; *P < 0.05, **P < 0.01.

Several ANO1 isoforms are generated by alternative splicing^26^,^27^. To determine the subcellular localization of ANO1 isoforms, such as ANO1(*0*), ANO1(*a*), ANO1(*ac*), and ANO1(*abc*), we constructed different human ANO1 isoforms tagged with green fluorescent protein (GFP) at the N-terminus and expressed the isoforms with DsRed-Mem, a plasma membrane marker, in HEK293T cells (Supplementary Fig. 1). ANO1(*a*), ANO1(*ac*), and ANO1(*abc*) were highly localized at the plasma membrane of transfected HEK293T cells (indicated by yellow color in the merged images). By contrast, ANO1(*0*), which has no *segment a, b,* or *c*, showed much lower expression at the plasma membrane. High expression of ANO1(*a*), ANO1(*ac*), and ANO1(*abc*) at the plasma membrane was also indicated by line scan analysis of images. The localization of ANO1 isoforms containing *segment a* at the plasma membrane raises the possibility that *segment a* of ANO1 contributes to the surface expression of these channels. To test this possibility, we constructed GFP-tagged ANO1(*abc*) and GFP-tagged ANO1(*abc*)-*Δa* (i.e., ANO1(*abc*) lacking *segment a*) to examine the involvement of *segment a* in the surface expression of ANO1 (Fig. 1a). Surface biotinylation of transfected HEK293T cells indicated that the surface expression of ANO1(*abc*)-*Δa* was markedly decreased compared with that of ANO1(*abc*) (Fig. 1b). In transfected HEK293T cells, GFP-ANO1(*abc*) was highly localized at the plasma membrane, whereas GFP-ANO1(*abc*)-Δ*a* was rarely found at the plasma membrane (Fig. 1c). A significantly higher expression of ANO1(*abc*) at the plasma membrane was indicated by a larger Pearson’s correlation coefficient between ANO1 and DsRed-Mem than between ANO1(*abc*)-Δ*a* and DsRed-Mem in HEK293T cells (Fig. 1d). The current-voltage (I-V) relationship in cells with ANO1(*abc*) showed sizable outward and inward currents (Fig. 1e, red trace). By contrast, cells with ANO1(*abc*)*-Δa* showed negligible currents (Fig. 1e, blue trace). The current densities of cells with ANO1(*abc*) and ANO1(*abc*)*-Δa* measured at +80 mV also showed that *segment a* is critical for ANO1 channel activity (Fig. 1f). This I-V relationship is consistent with the one in a previous study^28^. Taken together, these results indicate that *segment a* has a pivotal role in the surface expression and channel activity of ANO1.

### 14-3-3γ associates with ANO1 in heterologous systems

**Figure 2:**
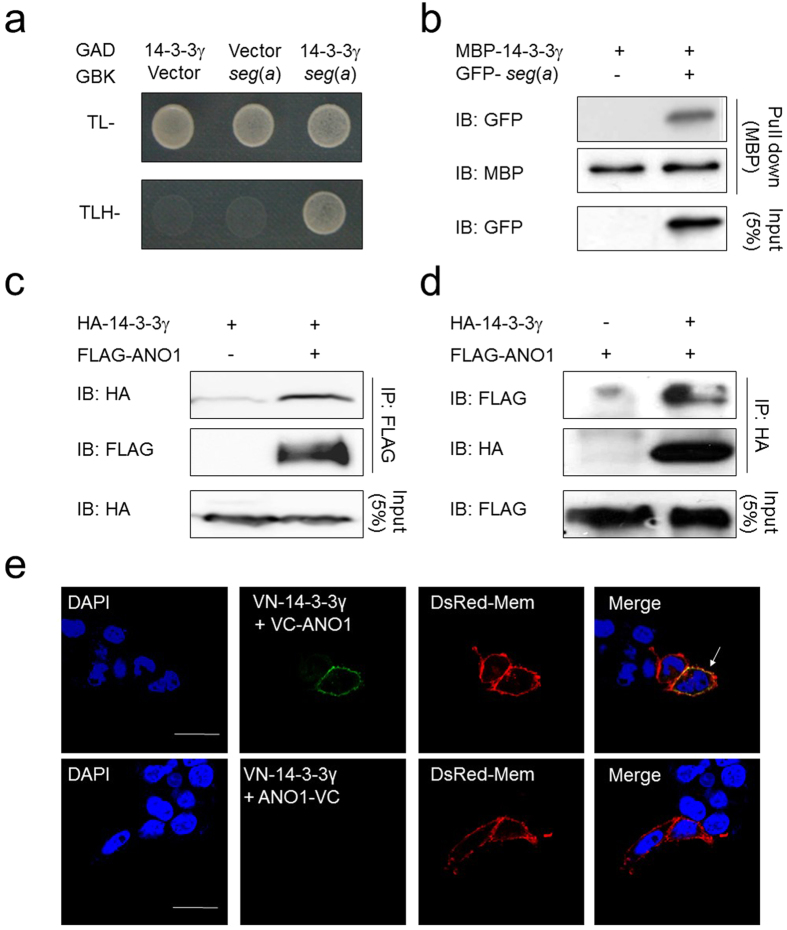
14–3–3γ directly binds to *segment a* of ANO1. (**a**) Y2H assay showed that *segment a* of ANO1 physically interacts with 14-3-3γ. (**b**) MBP pull-down assay showed that MBP-14-3-3γ binds to GFP-ANO1 *seg*(*a*). (**c,d**) Reverse Co-IP of full-length FLAG-ANO1 and HA-14-3-3γ confirmed their interaction. Western blot images (**b–d**) were cropped for clarification. Full blot images were included in supplementary information. (**e**) BiFC assay was performed in VN-14-3-3γ-transfected HEK293T cells with VC-ANO1 or ANO1-VC. Fluorescent signals provide evidence of the proximity of the two proteins at the plasma membrane (DsRed-Mem) as indicated by the yellow color (white arrow). Scale bars, 10 μm.

Based on our observation that *segment a* is critical for the surface expression of ANO1, we hypothesized that its surface expression may require certain factors bound to *segment a*. To identify potential interacting partners of *segment a*, we performed conventional Y2H screening using *segment a* as bait. Among positive clones isolated from Y2H screens, we identified human 14-3-3γ as a binding protein for *segment a*. We next confirmed an interaction between full-length cDNAs of 14-3-3γ and *segment a* by showing a positive yeast colony in Y2H under the non-permissive condition in yeast media without Thr, Leu, and His (TLH^−^), whereas no yeast colony was observed for the empty vector (Fig. 2a). A pull-down assay using maltose binding protein (MBP) also confirmed the interaction between 14-3-3γ and *segment a* (Fig. 2b). A co-immunoprecipitation (Co-IP) experiment clearly showed that *segment a* specifically bound to 14-3-3γ among seven 14-3-3 isoforms (Supplementary Fig. 2a).

To examine the interaction between 14-3-3γ and ANO1(*abc*) (referred to as ANO1 from here on) in a mammalian system, we constructed FLAG-tagged ANO1 (FLAG-ANO1) and hemagglutinin (HA)-tagged 14-3-3γ (HA-14-3-3γ) vectors and co-expressed them in HEK293T cells. We then performed Co-IP with cell lysates using anti-FLAG and anti-HA antibodies for IP or immunoblotting, respectively. We found that HA-14-3-3γ strongly associated with FLAG-ANO1 (Fig. 2c), and another Co-IP experiment performed using an opposite combination of antibodies showed the same result (Fig. 2d). Next, we examined the interaction between 14-3-3γ and ANO1 at the single-cell level to verify the subcellular location of this interaction. To do this, we used the bimolecular fluorescence complementation (BiFC) technique^27^, which allows visualization of two independent proteins in close spatial proximity. We constructed variants of 14-3-3γ and ANO1, whose N- and C-termini were fused with complementary halves of split Venus fluorescent protein, either the N-terminal half (VN) or the C-terminal half (VC), and co-transfected both into HEK293T cells (Fig. 2e). When the split Venus halves were in complementary positions in each subunit (VN-14-3-3γ + VC-ANO1), strong fluorescence was detected at the plasma membrane (Fig. 2e, upper panel). However, when the split Venus halves were fused at different positions (VN-14-3-3γ + ANO1-VC), no fluorescence was detected (Fig. 2e, lower panel). When VN-14-3-3γ and VC-ANO1 (or ANO1-VC) were separately expressed, no fluorescence was detected (Supplementary Fig. 2b). These results strongly suggest that an association between 14-3-3γ and ANO1 occurs at the plasma membrane of live mammalian cells.

### 14-3-3γ enhances the cell surface expression of ANO1 in heterologous systems

**Figure 3:**
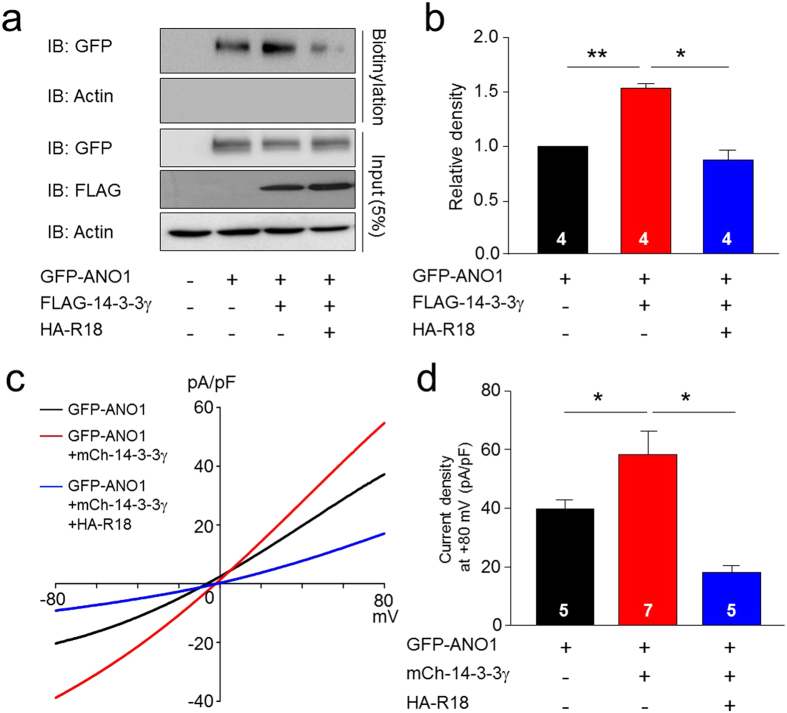
The surface expression of ANO1 was enhanced by 14-3-3γ. (**a**) Cell surface biotinylation results from membrane protein fractions from HEK293T cells transfected with GFP-ANO1, FLAG-14-3-3γ, and/or HA-R18. Western blot images were cropped for clarification. Full blot images were included in supplementary information. (**b**) Bar graph showing densitometric quantification of (**a**). Data are expressed as mean ± SEM; *P < 0.05, **P < 0.01. (**c**) Representative traces of whole-cell recordings of COS7 cells transfected with GFP-ANO1, 14-3-3γ, and/or HA-R18. (**d**) Summary bar graph showing normalized current density as in (**c**) at +80 mV. Data are expressed as mean ± SEM; *P < 0.05.

As 14-3-3 proteins affect the surface expression of several membrane proteins, including ion channels^29^,^30^, we next examined the effect of 14-3-3γ on the cell surface expression of ANO1. Both surface biotinylation (Fig. 3a,b) and luminescence-based cell surface assays (Supplementary Fig. 3a,b) indicated that the surface expression of ANO1 was markedly increased in the presence of 14-3-3γ in HEK293T cells. Additionally, R18, a peptide inhibitor of 14-3-3 proteins, significantly attenuated the enhancing effect of 14-3-3γ on the surface expression of ANO1 (Fig. 3a,b and Supplementary Fig. 3b). Consistent results were obtained from whole-cell patch clamp recordings. The presence of 14-3-3γ enhanced ANO1-mediated currents, and this effect was dramatically inhibited by R18 co-expression (Fig. 3c,d). In addition, Co-IP experiments (Supplementary Fig. 3c) and surface biotinylation (Supplementary Fig. 3d) showed that the association between ANO1, 14-3-3γ, and the 14-3-3γ-mediated surface expression of ANO1, were dramatically inhibited by the presence of R18 and *segment a*, indivisually. The *segment a* is critical for 14-3-3γ-mediated enhancement of ANO1 activity since 14-3-3γ also enhanced the channel activity of ANO1(*a*), which has only *segment a* (Supplementary Fig. 3e,f). These results suggest that 14-3-3γ enhances both the cell surface expression and channel activity of ANO1 isoforms containing *segment a*.

### Thr9 within *segment a* is critical for 14-3-3γ binding and surface expression of ANO1

Most 14-3-3 binding proteins have consensus binding sequences capable of mediating phosphorylation-dependent interactions with 14-3-3 proteins^31^. Because the surface expression of ANO1 was clearly enhanced by protein-protein interaction between 14-3-3γ and *segment a* of ANO1, we searched for possible binding sites for 14-3-3γ within *segment a* of ANO1 using the Scansite web program (http://scansite.mit.edu/motifscan_seq.phtml). We found one putative site on *segment a* for 14-3-3γ binding (^5^EKYSpTLP^11^). Indeed, phosphorylation of the Thr9 of ANO1 was enumerated at the Phosphosite database (http://www.phosphosite.org).

**Figure 4:**
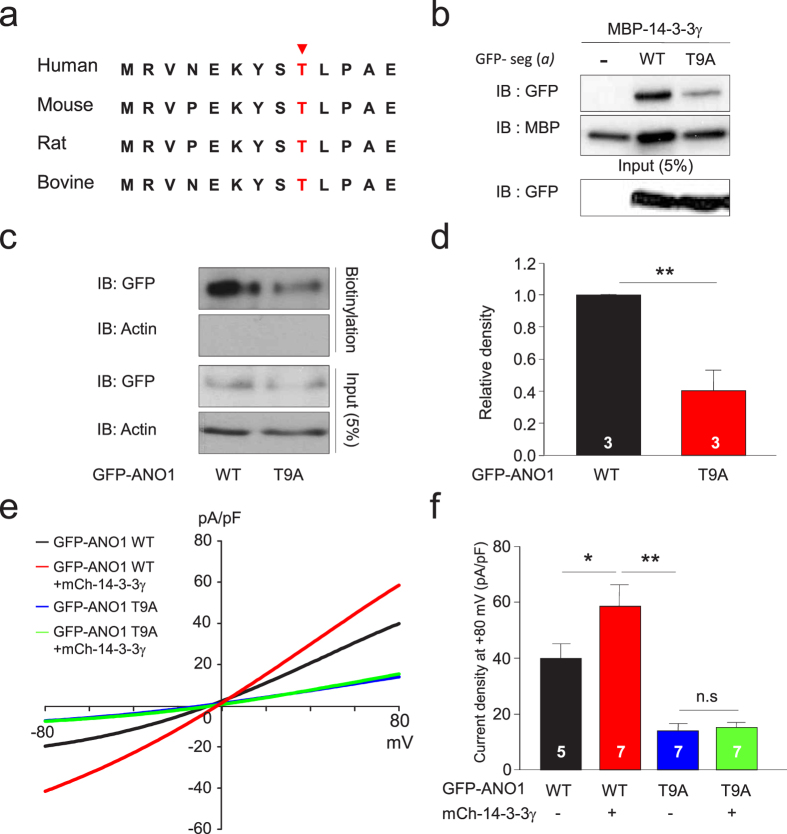
Thr9 in *segment a* of ANO1 is required for binding to 14-3-3γ. (**a**) Alignment of amino acid sequence of 14-3-3 binding motif in human, mouse, rat, and bovine ANO1. The red arrow indicates a predicted residue for phosphorylation-dependent 14-3-3 binding (http://scansite.mit.edu/motifscan_seq.phtml). (**b**) MBP pull-down assay showed that *seg*(*a*)-T9A bound to 14-3-3γ more poorly than *seg*(*a*) WT. (**c**) Cell surface biotinylation results from membrane protein fractions from HEK293T cells transfected with GFP-ANO1 WT or GFP-ANO1-T9A. Western blot images **(b,c**) were cropped for clarification. Full blot images were included in supplementary information. (**d**) Normalized bar graph showing data obtained from three independent experiments as in (**c**). Data are expressed as mean ± SEM; **P < 0.01. (**e**) Representative traces of whole-cell recordings in COS7 cells transfected with GFP-ANO1, GFP-ANO1-T9A, and/or mCh-14-3-3γ. (**f**) Summary bar graph showing normalized current density as in (g) at +80 mV. Data are expressed as mean ± SEM; n.s. = not significant, *P < 0.05, **P < 0.01.

Multiple sequence alignment of ANO1 proteins among four mammalian species showed that the phosphorylation-dependent binding site (^5^EKYSpTLP^11^) for 14-3-3γ is highly conserved (Fig. 4a). To determine whether phosphorylation of Thr9 of ANO1 is critical for the interaction between 14-3-3γ and *segment a* of ANO1, we replaced Thr with Ala in the ANO1 *segment a* to make *seg*(*a*)-T9A and assessed its ability to interact with 14-3-3γ. A MBP pull-down experiment revealed a dramatic reduction of protein-protein interaction between *seg*(*a*)-T9A and 14-3-3γ (Fig. 4b). Our Y2H assay also showed that *seg*(*a*)-T9A did not interact with 14-3-3γ (data not shown). We next explored the effect of this mutant on the surface expression of ANO1. Surface biotinylation and confocal imaging experiments showed that expression of ANO1-T9A in the plasma membrane was strikingly reduced compared to that of wild-type (WT) ANO1 in HEK293T cells (Fig. 4c,d and Supplementary Fig. 4a,b). Additionally, whole-cell recordings showed that ANO1-T9A-mediated currents were not affected by the presence of 14-3-3γ, whereas WT ANO1-mediated currents were dramatically increased (Fig. 4e,f). Therefore, we conclude that phosphorylation of Thr9 within *segment a* of ANO1 is critical for the 14-3-3γ binding, surface expression, and channel activity of ANO1.

### ANO1 is functionally expressed and associates with 14-3-3γ in glioblastoma cells

In addition to numerous human cancer tissues, a previous report showed that ANO1 is highly expressed in glioblastoma cells^22^. However, the channel activity of ANO1 has not been observed in these cells. Western blot analysis demonstrated protein expressions of ANO1 and 14-3-3γ in three glioblastoma cell lines, mouse primary astrocytes, and IM-PHFA cells, an immortalized human adult astrocyte cell line (Supplementary Fig. 5a). Compared to mouse primary astrocytes or IM-PHFA cells, high level of ANO1 expression was detected in the three glioblastoma cell lines (U251, T98G and U138). RT-PCR data showed that various ANO1 isoforms could be expressed in these glioblastoma cells. Especially, ANO1 isoforms containing the *segment a* are clearly expressed in these cells (Supplementary Fig. 5b).

**Figure 5:**
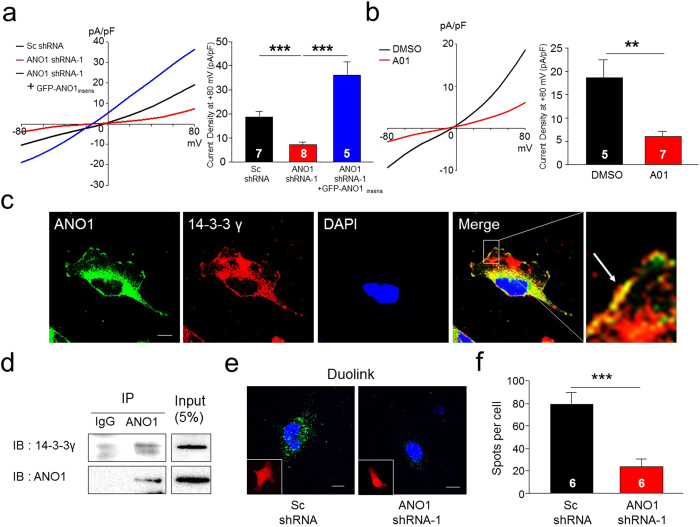
Endogenous ANO1 associates with 14-3-3γ in U251 cells. (**a**) Representative traces of whole-cell recordings of ANO1 in U251 cells transfected with Sc shRNA, ANO1 shRNA-1, and/or a GFP-ANO1-insensitive form of ANO1. (left panel). Summary bar graph showing normalized current density as in (left panel) at +80 mV (right panel). Data are expressed as mean ± SEM; ***P < 0.001. (**b**) Representative traces of whole-cell recordings of ANO1 in U251 cells treated with DMSO or A01 (an ANO1 inhibitor, 10 μM) (left panel). Data are expressed as mean ± SEM; **P < 0.01. Summary bar graph showing normalized current density as in (left panel) at +80 mV (right panel). (**c**) Representative immunocytochemical images of U251 cells stained with anti-ANO1 and anti-14-3-3γ antibodies. (**d**) Co-IP data show interactions between ANO1 and 14-3-3γ in U251 cells. Western blot images were cropped for clarification. Full blot images were included in supplementary information. (**e**) Representative images of Duolink PLA assay in U251 cells infected with adenovirus containing Sc shRNA and 14-3-3γ shRNA-1. Scale bar, 10 μm. (**f**) PLA signals were counted with Duolink Image tool software, and the average number of spots per cell is presented. Data are expressed as mean ± SEM; ***P < 0.001.

Next, to examine the functional expression of endogenous ANO1 channels and the association between ANO1 and 14-3-3γ in these glioblastoma cells, we constructed three different shRNAs against ANO1 and examined their silencing efficiency on ANO1 expression (Supplementary Fig. 5c). Among these ANO1 shRNAs, when either ANO1 shRNA-1 or shRNA-2 was transfected into U251 cells, Cl^−^ currents were significantly reduced (Supplementary Fig. 5d,e) compared with those of cells transfected with scrambled shRNA (Sc shRNA). We also made an ANO1 shRNA-1-insensitive form of ANO1 (ANO1_insens_, Supplementary Fig. 5f) for the rescue experiment. We next examined the silencing effect of ANO1 shRNAs on Cl^−^ currents in U251 cells. Expression of ANO1 shRNA-1 significantly reduced Cl^−^currents in U251 cells, whereas control Sc shRNA did not affect Cl^−^ currents (Fig. 5a,b). This silencing effect of ANO1 shRNA-1 became ineffective by co-transfection of ANO1 shRNA-1 with ANO1_insens_ (Fig. 5a). To confirm the importance of *segment a* for ANO1 surface expression, we generated several ANO1 shRNA-insensitive forms such as ANO1(*abc*), ANO1(*a*), ANO1-Δ*a*, and ANO1(*0*). The silencing effect of ANO1 shRNA-1 became recovered by ANO1(*a*)_insens_, only *segment a* containing ANO1 insensitive form (Supplementary Fig. 5g,h). In addition, the functional expression of ANO1 was confirmed by treatment with an ANO1 inhibitor, T16A_inh_-A01^32^ (A01) in U251 cell (Fig. 5b). We also measured ANO1 shRNA-1-sensitive Cl^−^ currents in two other glioblastoma cells, T98G and U138 (data not shown). These results indicate that ANO1 is functionally expressed in these glioblastoma cells.

Next, we determined whether the endogenous association of ANO1 and 14-3-3γ occurs in these cells. Immunocytochemical double-labeling showed strong co-localization of ANO1 and 14-3-3γ proteins in U251 cells (Fig. 5c). The enlarged images showed that ANO1 co-localized with 14-3-3γ at the plasma membrane. Co-IP data also showed a strong interaction between native ANO1 and 14-3-3γ in U251 cells (Fig. 5d). This interaction was further supported by a Duolink proximity ligation assay (PLA) (Fig. 5e and Supplementary Fig. 5i). A strong Duolink PLA signal from ANO1 and 14-3-3γ was observed in U251 cells expressing Sc shRNA but not in cells expressing ANO1 shRNAs. Quantification of PLA signals under these conditions is also shown in Fig. 5f and Supplementary Fig. 5j. Co-IP experiments also showed the similar results in both T98G and U138 cells (data not shown). Taking these findings together, we conclude that ANO1 is functionally expressed and associated with 14-3-3γ in glioblastoma cells such as U251, T98G and U138 cells.

### Deficiency of 14-3-3γ decreases the surface expression of ANO1 in U251 glioblastoma cells

**Figure 6:**
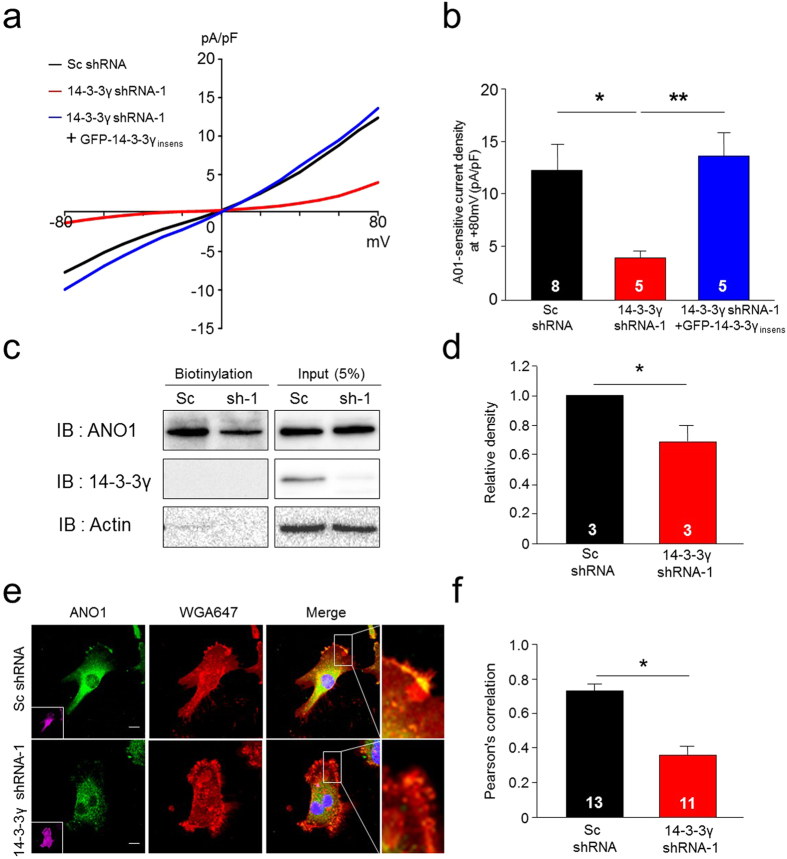
Deficiency of 14-3-3γ decreases the surface expression of ANO1 in U251 cells. (**a**) Representative traces of A01-sensitive currents in U251 cells transfected with Sc shRNA, 14-3-3γ shRNA-1, and/or a GFP-14-3-3γ-insensitive form of 14-3-3γ. (**b**) Summary bar graph showing normalized A01-sensitive current density as in (**a**) at +80 mV. Data are expressed as mean ± SEM; ***P < 0.001. (**c**) Cell surface biotinylation results from membrane protein fractions from U251 cells infected with adenovirus containing Sc shRNA or 14-3-3γ shRNA-1. Western blot images were cropped for clarification. Full blot images were included in supplementary information. (**d**) A normalized bar graph showing data obtained from three independent experiments as in (**c**). Data are expressed as mean ± SEM; *P < 0.05. (**e**) Representative immunocytochemical images of U251 cells transfected with Sc shRNA or 14-3-3γ shRNA-1. Scale bar, 10 μm. (**f**) Pearson’s correlation coefficients denoting covariance of fluorescence signals were calculated using Nikon software as in (**e**). Data are expressed as mean ± SEM; *P < 0.05.

As we found that 14-3-3γ enhances the surface expression of ANO1 in heterologous expression systems, we next investigated the effect of 14-3-3γ depletion on cell surface expression of endogenous ANO1 in glioblastoma cells. We developed three different shRNAs against 14-3-3γ and confirmed their knockdown efficiency using Western blot analysis (Supplementary Fig. 6a). Expression of both 14-3-3γ shRNA-1 and 14-3-3γ shRNA-2 significantly reduced ANO1-mediated currents in U251 cells, whereas control Sc shRNA did not affect ANO1-mediated currents (Supplementary Fig. 6b). In addition, 14-3-3γ shRNA-1 also reduced ANO1-mediated currents in T98G and U138 cells (Supplementary Fig. 6c,d). We also developed a 14-3-3γ shRNA-1-insensitive form of human 14-3-3γ (14-3-3γ_insens_) for rescue experiment (Supplementary Fig. 6e). As shown in Fig. 6a,b, 14-3-3γ shRNA-1 significantly reduced ANO1-mediated chloride currents in U251 and this effect of 14-3-3γ shRNA-1 were fully rescued by co-transfection of 14-3-3γ shRNA with 14-3-3γ_insens_.

We next examined the effect of silencing 14-3-3γ on ANO1 surface expression in U251 cells. Immunocytochemistry showed that 14-3-3γ shRNAs caused a significant reduction of ANO1 on the plasma membrane in U251 cells (Fig. 6a,b and Supplementary Fig. 6f,g). Expression of 14-3-3γ shRNAs caused a loss of co-localization between ANO1 and fluorescent-labeled wheat germ agglutinin (WGA647), a dye that labels membrane glycoproteins (or glycolipids) on the plasma membrane. ANO1 highly co-localized with WGA647 in Sc shRNA-treated cells (Fig. 6a and Supplementary Fig. 6f, upper panel), whereas ANO1 rarely co-localized with WGA647 in 14-3-3γ shRNA-transfected cells (Fig. 6a and Supplementary Fig. 6f, lower panel). Comparison of Pearson’s correlation coefficients showed that ANO1 expression at the plasma membrane was significantly reduced by depletion of 14-3-3γ (Fig. 6b and Supplementary Fig. 6g). Surface biotinylation assay with U251 cells also showed that infection with adenovirus containing 14-3-3γ shRNA-1 caused a significant reduction in the surface expression of ANO1 compared with adenovirus containing Sc shRNA, without affecting total ANO1 protein levels (Fig. 6c,d). These data demonstrated depletion of 14-3-3γ reduced the ANO1-mediated currents and ANO1 surface expression in glioblastoma cells.

### Deficiency of ANO1 or 14-3-3γ suppresses migration and invasion of U251 glioblastoma cells

**Figure 7:**
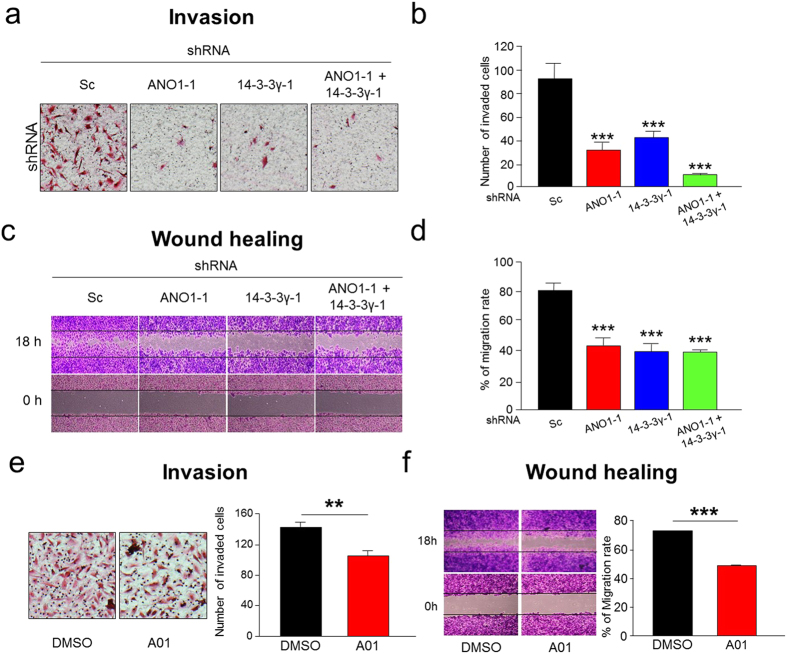
Inhibition of channel activity and trafficking of ANO1 in U251 cells decreases invasion and migration. (**a**) Representative images of collagen-coated transwell invasion assay in U251 cells infected with Sc shRNA, 14-3-3γ shRNA-1, and/ or ANO1 shRNA-1. (**b**) Normalized bar graphs showing data obtained from three independent experiments as in (**a**). Data are expressed as mean ± SEM; ***P < 0.001. (**c**) Representative images of wound-healing assay in U251 cells infected with Sc shRNA, 14-3-3γ shRNA-1, and/ or ANO1 shRNA-1. (**d**) Normalized bar graphs showing data obtained from three independent experiments as in (**c**). Data are expressed as mean ± SEM; ***P < 0.001. (**e)** Representative images (left panel) of collagen-coated transwell invasion assay in U251 cells treated with DMSO or A01 (10 μM for 18 h) Normalized bar graphs (right panel) showing data obtained from three independent experiments as in (left panel). Data are expressed as mean ± SEM; **P < 0.01. (**f**) Representative images (left panel) of wound-healing assay in U251 cells treated with DMSO or A01 (10 μM for 18 h) Normalized bar graphs (right panel) showing data obtained from three independent experiments as in (left panel). Data are expressed as mean ± SEM; ***P < 0.01.

Because ANO1-mediated currents were reduced by depletion of 14-3-3γ in U251, T98G, and U138 glioblastoma cells, we examined whether gene silencing by adenovirus containing the shRNA against 14-3-3γ or ANO1 affect characteristics of cancer cells, such as migration and invasion. As expected, depletion of 14-3-3γ or ANO1 in U251 cells resulted in a significant reduction in invasiveness (collagen-coated transwell invasion assay) of U251 cells compared with that of Sc shRNA-infected cells (Fig. 7a,b and Supplementary Fig. 7a,b). In addition, invasiveness of these cells were additively suppressed by co-expression of both shRNAs against 14-3-3γ and ANO1. Depletion of 14-3-3γ or ANO1 in T98G and U138 cells also showed similar results (Supplementary Fig. 7a,b). We also examined the silencing effect with the shRNA against 14-3-3γ or ANO1 on migration (wound-healing assay) in U251 cells (Fig. 7c,d). The wound area recovered rapidly (within 18 h) in Sc shRNA-infected cells. However, wound closure was delayed in 14-3-3γ or ANO1 shRNA-infected cells when examined at the same time point. There was no additive effect in wound healing assay when both shRNAs were co-infected. Furthermore, A01, an ANO1 selective inhibitor, also inhibited invasiveness and migration of U251 cells (Fig. 7e,f). Taken together, these results strongly suggest that suppression of ANO1 surface expression inhibits cancer progression of human glioblastoma cells (Supplementary Fig. 7e).

## Discussion

Alternative splicing generates several ANO1 isoforms that include or skip *segments a*, *b*, *c*, or *d*. However, the detailed roles of these *segments* are poorly understood. The present study uncovers the pivotal role of *segment a* in ANO1 expression at the plasma membrane by identifying 14-3-3γ as a binding protein. We provided evidence that 14-3-3γ directly bound at a conserved binding motif within *segment a* of ANO1 channels. This binding between 14-3-3γ and *segment a* of ANO1 depended on putative phosphorylation of Thr9 of ANO1. Furthermore, we demonstrated oncogenic roles of 14-3-3γ-mediated ANO1 surface expression in U251 glioblastoma cells.

A large number of ANO1-interacting proteins were recently discovered using the stable isotope labeling of amino acids in culture (SILAC) proteomics technique in ANO1 over-expressing HEK293 cells^33^. A total of 93 interacting proteins were found for ANO1, and interactions between ANO1 and selected proteins such as ezrin, radixin, and moesin were validated in salivary gland epithelial cells. These putative interacting proteins for ANO1 are useful for understanding the regulatory network of ANO1, although the detailed functions of each interaction should be examined. However, it is still possible that additional binding proteins are yet to be found in particular cell types that endogenously express ANO1. Indeed, in the present study, we found a novel binding protein for ANO1, 14-3-3γ, which interacts with *segment a* of ANO1.

ANO1 has several isoforms such as (*a*), (*ac*), (*abc*), (*acd*), (*abcd*), and (*0*) based on different combinations of *segments a, b, c,* and *d*. A previous study shows that ANO1 isoforms containing *segment a* are initiated by the same promoter^26^ and generated from the same primary transcript via alternative splicing^5^. By contrast, the skipping of *segment a* results from an alternative promoter, leading to the generation of ANO1(*0*)^26^. Because we found that 14-3-3γ interacted with the consensus binding motif within *segment a*, and surface expression of ANO1(*abc*) was enhanced by 14-3-3γ, it is also possible that the surface expression of other ANO1 isoforms containing *segment a* could be enhanced by 14-3-3γ interaction. Indeed, we found that the surface expression of ANO1(*a*) and ANO1(*ac*) was also increased by 14-3-3γ (data not shown). Although ANO1(*0*) showed the poor expression at the plasma membrane compared with other ANO1 isoforms, a small population of this isoform was detected at the plasma membrane. Based on these observations, we cannot exclude the possibility that other regulatory mechanisms of the surface expression of ANO1 might exist, although a 14-3-3γ-dependent mechanism seems to be dominant.

Most 14-3-3 binding proteins have consensus binding sequences capable of mediating phosphorylation-dependent interactions with 14-3-3 protein^31^. In this study, we demonstrated that ANO1 has one mode II binding site (^5^EKYSpTLP^11^) within *segment a,* and Thr9 is critical for specific 14-3-3γ binding and surface expression of ANO1 (Fig. 5). 14-3-3 proteins, including 14-3-3γ, exist as either homo- or hetero-dimers, and each monomer contains a ligand-binding groove allowing each 14-3-3 dimer to bind to two separate binding sites^31^. As ANO1 isoforms function as dimers^8^,^34^,^35^, functional ANO1 channels containing *segment a* may have two binding sites for 14-3-3γ. Therefore, the surface expression of ANO1 channels containing *segment a* might be regulated by phosphorylation-dependent binding with 14-3-3γ dimer as multiple heterodimeric complexes.

Most 14-3-3 binding to cargo proteins requires phosphorylation of the cargo, but the relevant kinases are unknown in most cases. As the present study shows that 14-3-3γ enhances surface expression of ANO1 via phosphorylation-dependent binding at the Thr9 residue of ANO1, the identification of physiologically relevant kinases is critical for understanding the mechanism of 14-3-3γ-mediated ANO1 trafficking. To understand the detailed mechanism of ANO1 regulation, the physiological functions of ANO1 in various tissues, the oncogenic functions of ANO1 in diverse cancer cells, and the specific kinases that phosphorylate at Thr9 residue within *segment a* of ANO1 should be examined in future studies.

In addition to the role of 14-3-3γ in channel trafficking^30^, previous studies demonstrated the involvement of 14-3-3γ in tumorigenesis. 14-3-3γ has been shown to be one of key genes involved in osteosarcoma and head and neck squamous cell carcinoma^36^,^37^. Overexpression of 14-3-3γ has been reported in non-small cell lung cancer, promyelocytic leukemia and glioblastoma^38^,^39^,^40^. As an oncogene, 14-3-3γ stimulates MAPK and PI3K signaling^41^. Since knockdown of 14-3-3γ inhibits invation and migration of glioblastoma cells in our study (Fig. 6a–d and Supplementary Fig. 7a,b), 14-3-3γ also seems to have a oncogenic role in gliablastoma cells. Therefore, 14-3-3γ-mediated signaling pathway should be examined in detail in further study.

Altered expressions and their dysfunctions of various ion channels and transporters have been implicated in diverse types of cancer^42^,^43^. Especially, chloride channels such as CLCA1, CLCA2 and CLCA4 are downregulated in human colorectal tumors^44^,^45^, in contrast, CLIC1 in gastric cancer and CLIC3 in pancreatic cancer have been shown to be upregulated^46^. Therefore, chloride channels could be either tumor suppressors or tumor-promoting factors (if not oncogenes themselves) depending on the origins and types of cancers. Interestingly, glioblastoma-associated CLIC1, an intracellular chloride channel has been shown to be involved specifically in tumorigenesis by transporting it to the plasma membrane and functioning as an ion channel^47^,^48^.

Chloride channels are critical in maintaining cell volume, and therefore, play important roles in migration and invasion of cancer cells, especially for glioma^49^. Non-specific blockers of chloride channels have been shown to reduce migration of glioma cells^50^. ANO1 has been shown to be upregulated in glioblastoma^22^. In consistent with our results in glioblastoma cells, A01, a specific ANO1 inhibitor, has been shown to inhibit the proliferation of CFPAC pancreatic cancer cells and colorectal cancer cell lines^51^,^52^. A specific knockdown of ANO1 also suppressed cell proliferation, migration, and invasion of colorectal cancer cells and glioblastoma cells^22^,^52^. Taken together with the effects of A01 and shRNA in the present study, we believe that loss of ANO1 channel activity is directly responsible for migration and invasion of glioblastoma^21^,^22^,^52^. NF-κB signaling has been shown to be activated in ANO1-overexpressing glioma, and the expressions of NF-κB-mediated genes are involved in proliferation, migration and invasion of glioma cells^22^. As our results clearly showed that ANO1 surface expression is enhanced by 14-3-3γ binding, and that gene silencing of ANO1 or 14-3-3γ suppresses migration and invasion of glioblastoma cells, 14-3-3γ-mediated ANO1 surface expression may play a key role in tumorigenesis by activating MAPK signaling or NF-κB signaling, which should be investigated in a future study.

In conclusion, our finding of the regulation of ANO1 surface expression by 14-3-3γ binding establishes a new role for *segment a* of ANO1 and indicates that 14-3-3γ-mediated ANO1 surface expression has an important role in the cancerous progression of glioblastoma cells. This novel regulatory mechanism of ANO1 should prove useful for further efforts to understand its functional roles in physiological conditions. Finally, this finding may help the development of effective therapeutic targets for various ANO1-mediated cancers, including glioblastoma.

## Methods

### Chemical

T16A_inh_-A01 was purchased from Sigma-Aldrich.

### Cell culture and transfection

HEK293T, U251, U138 and T98G cells were cultured in DMEM (Gibco). COS7 cells were maintained in RPMI (Gibco). All cells were maintained in medium containing 10% FBS (Gibco) and 100 units/ml penicillin-streptomycin (Gibco) at 37 °C with 5% CO_2_. Transfection of expression vectors was performed using Lipofectamine 2000 (Invitrogen) according to the manufacturer’s protocol.

### Construction of plasmids

cDNAs encoding full-length human ANO1 (GenBank Accession No. XM_011545125) and human 14-3-3γ (NM_012479.3) were obtained using a RT-PCR-based gateway cloning method (Invitrogen). Several mutants of ANO1 including ANO1(*abc*), ANO1(*ac*), ANO1(*bc*), ANO1(*0*), ANO1(*abc*)*-Δa*, ANO1 *segment a* (*seg*(*a*)), and ANO1 T9A were generated using full-length human ANO1 cDNA as a template via an EZchange^TM^ site-directed mutagenesis kit (Enzynomics). All constructs were subcloned into several vectors, including pDEST-FLAG-N, pDEST-HA-N, pDEST-GFP-N, and pDEST-mCherry-N, by gateway cloning (Invitrogen). The R18 peptide sequence (GVTQGALESTLDLEANMCL) was obtained using an EZchange^TM^ site-directed mutagenesis kit (Enzynomics).

### Construction of shRNAs, and adenovirus

The ANO1 and 14-3-3γ-shRNAs target sequences were; for ANO1 shRNA-1, 5′-GCATCCAGCTCAGCATCAT-3′ (1970–1990 nt); for ANO1 shRNA-2, 5′-CGTGTACAAAGGCCAAGTA-3′ (773–779 nt); for 14-3-3 γ-shRNA-1, 5′-ACGAGGACTCCTACAAGGAC-3′(635–654 nt); for 14-3-3 γ-shRNA-2, 5′-GATTAGGCCTGGCTCTTAACT-3′(515–535 nt). pSicoR-ANO1-shRNAs and pSicoR-14-3-3 γ-shRNAs were constructed using the EZchange^TM^ site-directed mutagenesis kit (Enzynomics) and confirmed by sequencing. For adenovirus production, For production of adenovirus, pSicoR cassette containing U6 promoter, shRNA sequences and CMV-mCherry flanked by loxP sites was transferred to pAd/CMV/V5-DEST^TM^ vector using a gateway system and concentrated virus was produced by Virapower^TM^ Adenoviral Gateway Expression Kit (Invitrogen), according to the manufacturer’s instructions.

### Analysis of ANO1 Splicing variants

Total RNA derived from U251, U138 and T98G cells was reverse-transcribed using RNA to cDNA EcoDry^TM^ (Clontech) and subsequently amplified by PCR with a set of primers specific for each alternatively spliced exon. Conditions for PCR were the following: for *segment a, segment b, segment c* and GAPDH, 94 °C for 3 min for the initial denaturation; 94 °C for 45 s, 58 °C for 45 s, and 72 °C for 1 min for 35 cycles; and 72 °C for 5 min for the final extension. We used the following sense and antisense primers: for *segment* *a*, forward (5′-GGCCTGTACTTCAGGGACG-3′) and reverse (5′-GCTCCAGCTCCAGGCCCGCC-3′); for *segment* *b*, forward (5′-CGGAGCACGATTGTCTATGA-3′) and reverse (5′-AACTCCATAGCGTGCCCACTCTTCG-3′); and for *segment* *c*, forward (5′-CTCTGTCTTCATGGCCCTC-3′) and reverse (5′-CTCCAAGACTCTGGCTTCGT-3′); for GAPDH, forward (5′-GTCTTCACCACCATGGAGAA-3′) and reverse (5′-GCATGGACTGTGGTCATGAG-3′).

### Y2H screening and assay

ANO1 *seg*(*a*) was ligated into the GAL4 DNA binding domain (BD), and 14-3-3γ was cloned into the activation domain (AD). To assess the protein-protein interaction between ANO1 and 14-3-3γ, BD/ANO1 *seg*(*a*) and AD/14-3-3γ were co-transformed into the yeast strain AH109, which was incubated in selection medium.

### Co-IP and immunoblotting

For Co-IP, the lysates of transfected HEK293T cells and U251 cells were prepared with lysis buffer (50 mM Tris–HCl, pH 7.4, 150 mM NaCl, 5 mM EDTA, and 0.1% Triton X-100) containing a protease-inhibitor cocktail (Roche) and mixed overnight at 4 °C with anti-HA (F-7; Santa Cruz Biotechnology), anti-FLAG (M-2; Sigma), or anti-ANO1 (ab53212; Abcam) antibodies. Immune complexes were incubated by binding to mixed protein A/G beads (Santa Cruz Biotechnology) for 1 h. For immunoblotting, protein samples were separated by 10% SDS-PAGE gel under the same experimental condition and then transferred to PVDF membranes. The blots were incubated overnight at 4 °C with anti-HA (1:1000; 3F10, Roche), anti-FLAG (1:1000; M-2, Sigma), anti-GFP (1:1000; B-2, Santa Cruz Biotechnology), or anti-14-3-3γ (1:1000; ab69592, Abcam) antibodies. Blots were then incubated with HRP-conjugated goat anti-mouse, anti-rat, or anti-rabbit IgG followed by detection of immunoreactivity with enhanced chemiluminescence (Amersham Biosciences).

### MBP pull-down assay

14-3-3γ was cloned into MBP vector and expressed and purified in bacteria. Briefly, BL-21 cells transformed with MBP-14-3-3γ were incubated with IPTG (1 mM) for 3 h, harvested by centrifugation, and then lysed by sonication. HEK293T cells were transfected with GFP-*seg*(*a*) or GFP-*seg*(*a*)*-T9A* and extracted using lysis buffer. Cell lysates were incubated with pre-immobilized MBP agarose beads (Elpis). After 2 h incubation at 4 °C, beads were washed with ice-cold PBS. Bound proteins were eluted with SDS sample buffer, and analyzed by western blotting.

### Cell surface biotinylation

For cell surface biotinylation, GFP-ANO1-transfected HEK293T and U251 cells were incubated at 4 °C and washed with PBS. Surface-expressed proteins were then biotinylated in PBS containing sulfo-NHS-SS-biotin (Pierce, Rockford, IL) for 30 min. After biotinylation, cells were washed with quenching buffer (100 mM glycine in PBS) and then washed with PBS. Cells were then lysed and incubated with high-capacity NeutrAvidin-agarose resin (ThermoScience). After three washes with lysis buffer, bound proteins were eluted by SDS sample buffer and subjected to western blot analysis.

### Imaging analysis and bimolecular fluorescence complement (BiFC) assay

For imaging analysis, HEK293T cells grown on coverslips were co-transfected with GFP-tagged ANO1 vectors and pDsRed-Mem (Clontech Laboratories). Cells were fixed in 4% paraformaldehyde (PFA) for 20 min at room temperature (RT). After washing with PBS, cells were observed under a Nikon A1 confocal microscope. To quantify co-localization in merged images, at least 10 cells were randomly selected and analyzed. Pearson’s correlation coefficients were computed using Nikon A1 confocal microscope software. The *r* values are between 0 and 1. A value of 1 means that perfect co-localization occurs, while a value of 0 means that no co-localization is seen. Fluorescence intensity was measured using line scan-based analysis in Nikon A1 confocal microscope software. For BiFC, ANO1 and 14-3-3γ were cloned into pBiFC-VN173 and pBiFC-VC155 vectors (Addgene). HEK293T cells were co-transfected with cloned BiFC vectors in all possible pairwise combinations. The next steps were the same as those for imaging analysis, and Venus fluorescence signals were observed by confocal microscopy.

### Immunocytochemistry

U251 cells growing on coverslips were transfected with Sc shRNA or 14-3-3 shRNAs and incubated for an additional 72 h. Cells were fixed in 4% PFA for 20 min at RT and then incubated with WGA647 conjugate (1:200; Thermo) at 4 °C for 15 min to preferentially label the plasma membrane. Cells were permeabilized with Triton X-100 (0.5% in PBS) and blocked with 5% BSA. Cells were incubated overnight at 4 °C with anti-ANO1 (1:100; ab64085, Abcam) or anti-14-3-3γ (1:100; ab118877, Abcam) antibodies. After washing, cells were incubated with DyLight 488- or 549-conjugated secondary antibodies (1:400; Jackson Labs) for 1 h at RT. Cells were washed, mounted, and observed under a Nikon A1 confocal microscope.

### Duolink PLA

Interactions between endogenous proteins were detected using a Duolink PLA kit (Bioscience; Detection Kit 488), according to the manufacturer’s instructions. The primary antibodies used for this assay were anti-ANO1 (1:100; ab64085, Abcam) and anti-14-3-3γ (1:100, ab118877, Abcam) antibodies. The PLA probe anti-rabbit minus binds to the anti-ANO1 antibody, whereas the PLA probe anti-mouse plus binds to the anti-14-3-3γ. U251 cells growing on coverslips were transfected with Sc shRNA or ANO1 shRNAs, and incubated for 72h. Cells were observed using a Nikon A1 confocal microscope

### Electrophysiological recording

Current-voltage (*I-V*) curves were obtained from COS-7 cells expressing ANO1 or ANO1 mutants or from transfected U251, T98G and U138 cells. Currents were measured by applying 1s duration voltage ramps from +100 to −100 mV (a holding potential of −70 mV) at 20–22 °C. For measuring Cl^−^ currents, recording electrodes (4–7 MΩ) were filled with (mM): 146 CsCl, 5 Ca-EGTA-NMDG, 8 HEPES, 2 MgCl2, and 10 sucrose (pH adjusted to 7.3 with CsOH). The standard bath solution contained (in mM): 150 NaCl, 10 HEPES, 3 KCl, 2 CaCl2, 2 MgCl2, and 5.5 glucose (pH adjusted to 7.3 with NaOH). Whole-cell currents were amplified using the Axopatch 200A patch clamp system. Data acquisition was controlled by pCLAMP 10.2 software (Molecular Devices).

### Wound-healing assay

Adenovirus infected U251 and naïve U251 cells were plated onto 24-well plates at a density of 2 × 10^5^ cells per well. After 24 h, cells were scraped with a SPLScar^TM^ Scratcher (SPL Life Sciences), and incubated in complete media for 24 h. Cells were stained with 0.1% crystal violet, and images were taken. The ratio of the remaining wound area was calculated relative to the initial wound area and normalized to that for Sc shRNA-infected cells.

### Cell invasion assay

Transwell invasion chambers (BD Biosciences) were used according to the manufacturer’s instructions, and 100 μl collagen mix was used to coat the membrane. Adenovirus infected U251, U138, T98G and naïve U251 cells were plated onto a transwell membrane insert at a density of 1 × 10^5^ cells per well in 100 μl DMEM. The lower chambers were filled with 500 μl DMEM. The transwells were then incubated for 24 h to allow cells to migrate. At the end of incubation, cells on the upper side of the insert filter were completely removed using a cotton swab, and cells that had invaded through the collagen-coated membrane were fixed in 4% PFA and stained with hematoxylin and eosin. For quantification, cells were counted under a microscope in eight random fields at 20×magnification. The experiment was repeated in triplicate.

### Statistical analysis

Numerical data are presented as mean ± SEM. The significance of data for comparison was assessed by Student’s two-tailed unpaired t-test and significance levels were given as: *P < 0.05, **P < 0.01 and ***P < 0.001.

## Additional Information

**How to cite this article**: Lee, Y.-S. *et al.* Suppression of 14-3-3γ-mediated surface expression of ANO1 inhibits cancer progression of glioblastoma cells. *Sci. Rep.* **6**, 26413; doi: 10.1038/srep26413 (2016).

## Supplementary Material

Supplementary Information
