# Supplementary material for: Suppression of 14-3-3γ-mediated surface expression of ANO1 inhibits cancer progression of glioblastoma cells
Source: Sci Rep. 2016 May 23;6:26413. doi: 10.1038/srep26413 (PMC4876403; doi:10.1038/srep26413)
Supplement: Supplementary Information [file srep26413-s1.pdf]

Supplementary Information

Suppression of 14-3-3γ-mediated surface expression of ANO1 inhibits cancer progression of glioblastoma cells

Young Sun Lee[1,2,3], Jae Kwang Lee[4], Yeonju Bae[1], Bok-Soon Lee[5], Eunju Kim[1,3], Chang-Hoon Cho[1], Kang-Hyun Ryoo[1], Jiyun Yoo[6], Chul-Ho Kim[5], Gwan-Su Yi[7,] Seok-Geun Lee[8,] C. Justin Lee[3,4], Sang Soo Kang[2], Eun Mi Hwang[3,4*], and Jae-Yong Park[1*]

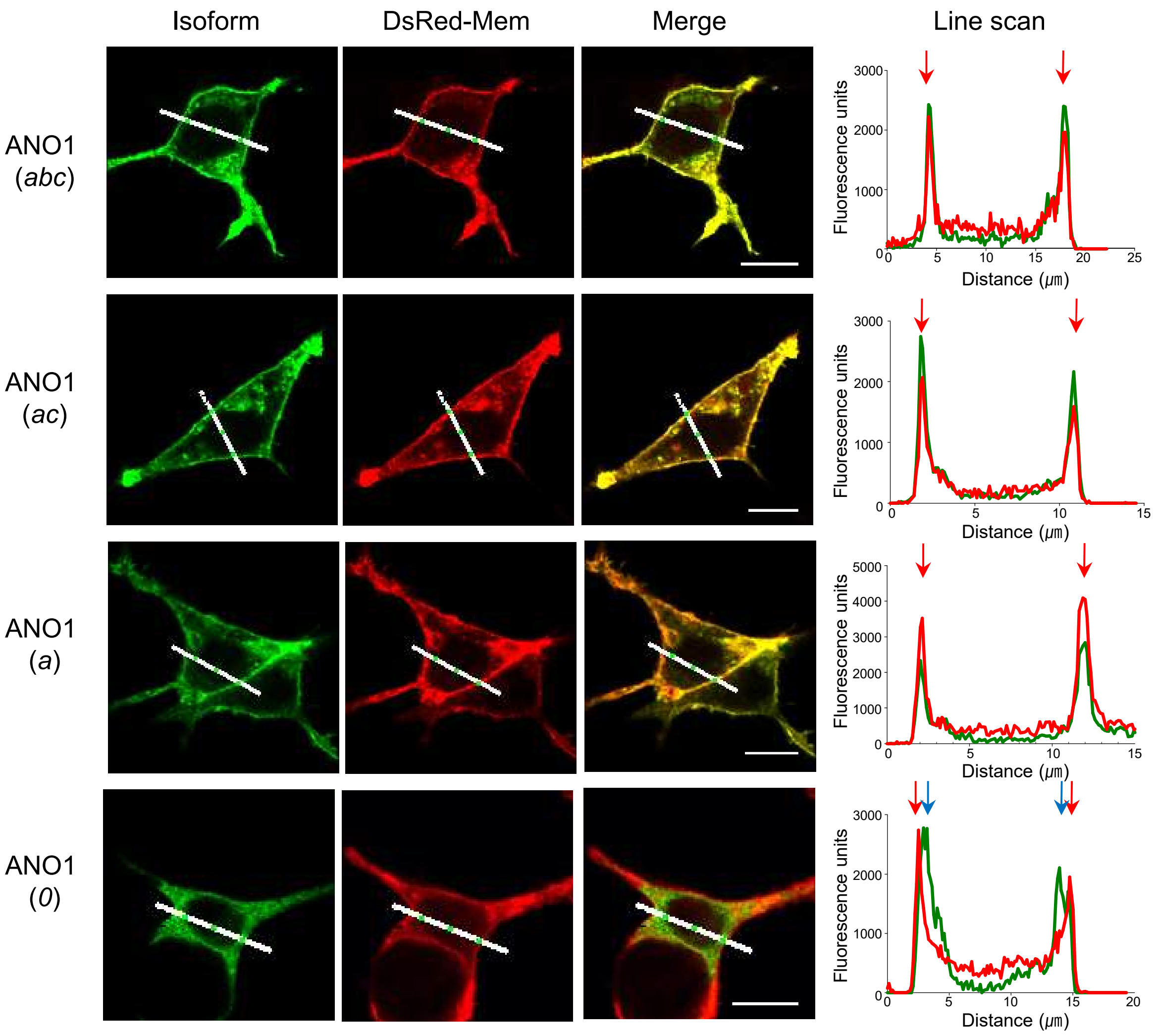

Supplementary Figure 1. Subcellular localization of ANO1 isoforms. HEK293T cells were transfected with GFP-ANO1*(abc)*, GFP-ANO1*(ac)*, GFP-ANO1*(a),* or GFP-ANO1*(0)* and the plasma membrane marker, DsRed-Mem. Localization at the plasma membrane was evaluated from plots of normalized fluorescence intensity of ANO1 isoforms and DsRed-Mem using the 'line scan' feature of Nikon software. The colored arrows mark the maximal intensity of each signal. Scale bars, 10 μm.

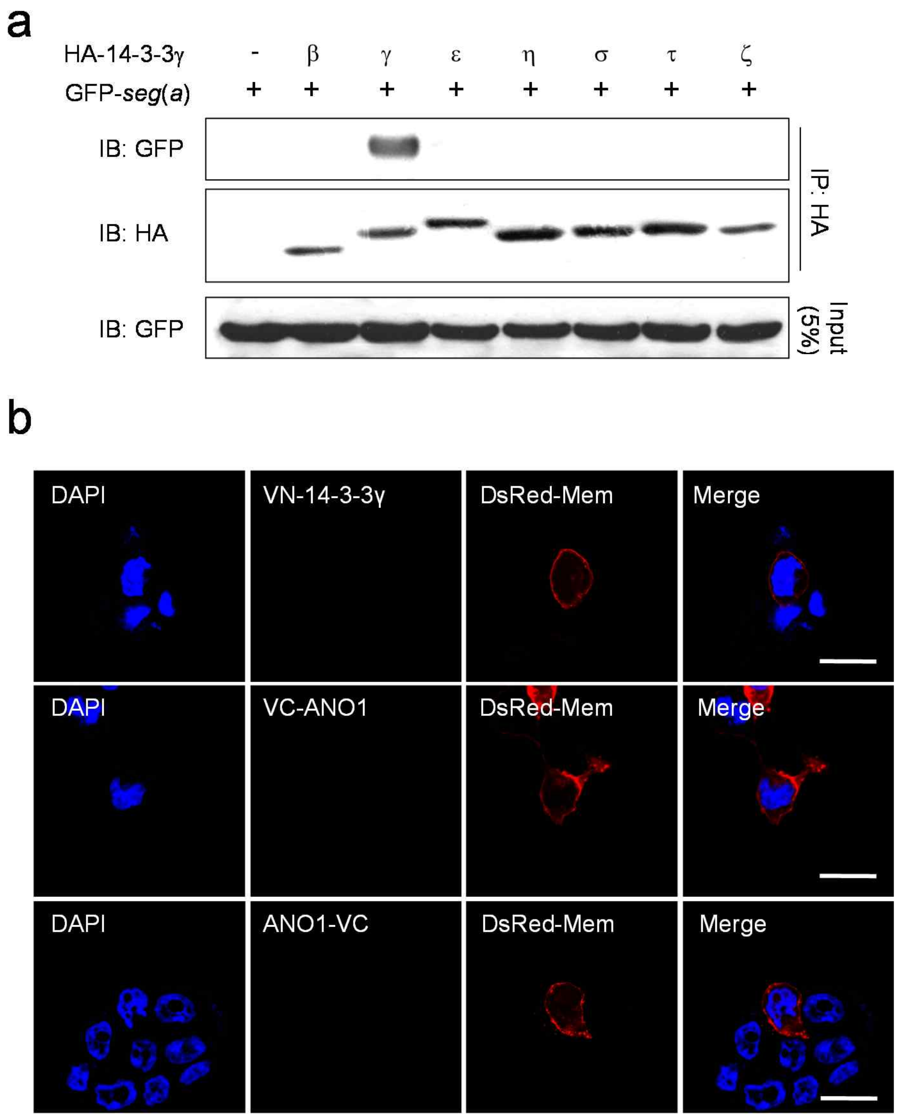

Supplementary Figure 2. ***Segment a*** **of ANO1 specifically interacts with 14-3-3γ.** (a) Co-IP with GFP-*seg(a)* and seven HA-14-3-3 isoforms showed the specific interaction of 14-3-3γ with ANO1 *segment a* in HEK293T cells. (b) Negative control for BiFC experiments. No signal of Venus fluorescence was detected 24 h after co-transfection of one half of split Venus and DsRed-Mem. The nuclei were indicated by DAPI staining. Scale bar, 10 μm.

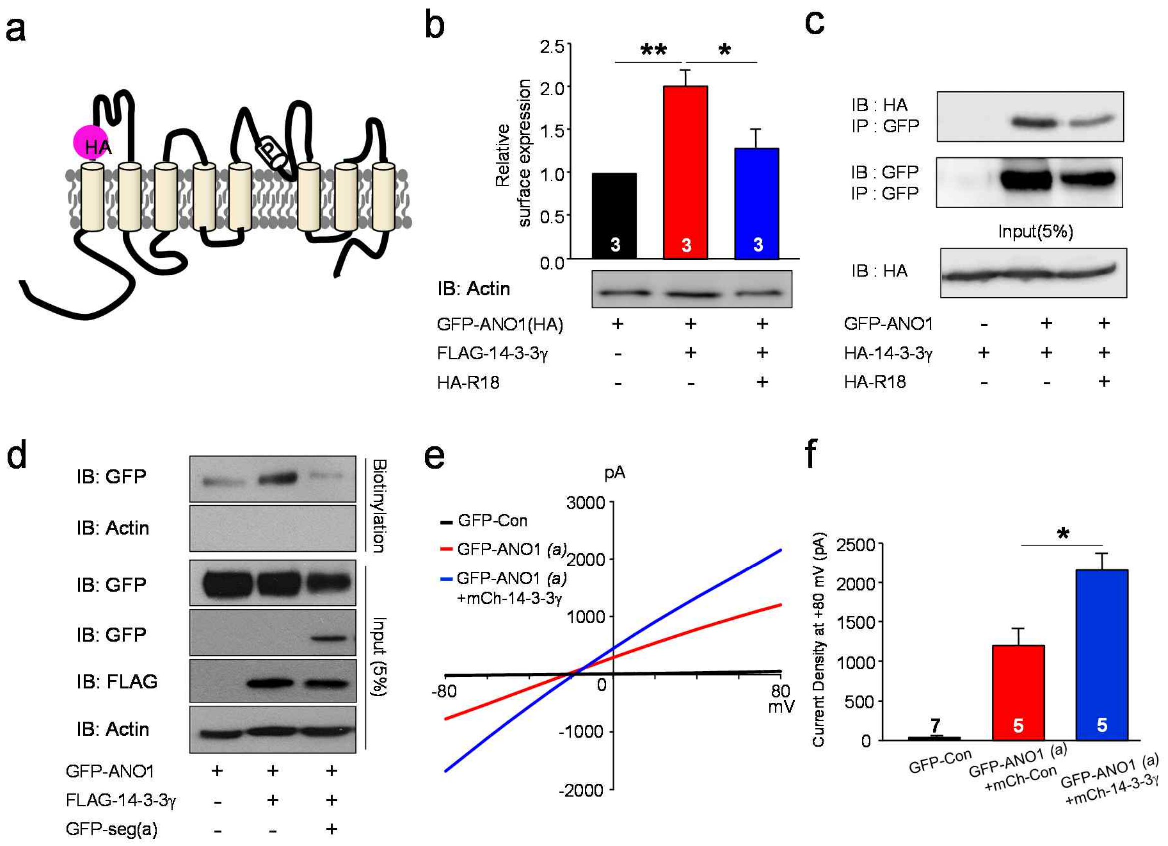

Supplementary Figure 3. 14-3-3γ-mediated surface expression of ANO1 is blocked by R18 peptide and *segment a* of ANO1. (a, b) Quantification of ANO1(HA) cell surface expression in the absence or presence of additional 14-3-3γ or HA-R18 using an anti-HA antibody. Cell surface expression of ANO1 was normalized to the average of the ANO1 expression-only condition. The number on each bar indicates the *n* for each condition. Data were obtained from three independent experiments and are expressed as mean±SEM; **P<0.01, *P<0.05. (c) Co-IP data from HEK293T cells transfected with GFP-ANO1, 14-3-3γ, or GFP-ANO1 *seg(a)*. (d) Cell surface biotinylation assay of HEK293T cells transfected with GFP-ANO1, 14-3-3γ, or GFP-ANO1 *seg(a)*. (e) Representative traces of whole-cell recordings of HEK293T cells transfected with GFP-ANO1 *(a)* and/or mCh-14-3-3γ. (f) A summary bar graph showing normalized current density as in (e) at +80 mV. Data are expressed as mean±SEM; *P<0.05.

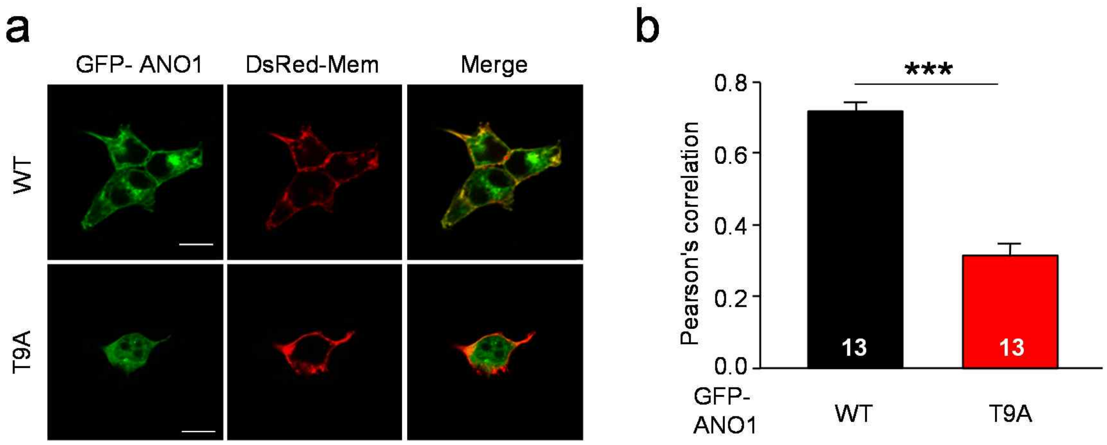

Supplementary Figure 4. Thr9 is critical for ANO1 expression at the plasma membrane. (a) Subcellular localization showed that ANO1-T9A overlapped with DsRed-Mem more poorly than ANO1-WT in HEK293T cells. Scale bars, 10 μm. (b) Bar graphs show Pearson's correlation coefficients denoting covariance of fluorescence signals calculated using Nikon software. The number on each bar indicates the *n* for each condition. Data are expressed as mean±SEM; ***$P<0.001$.

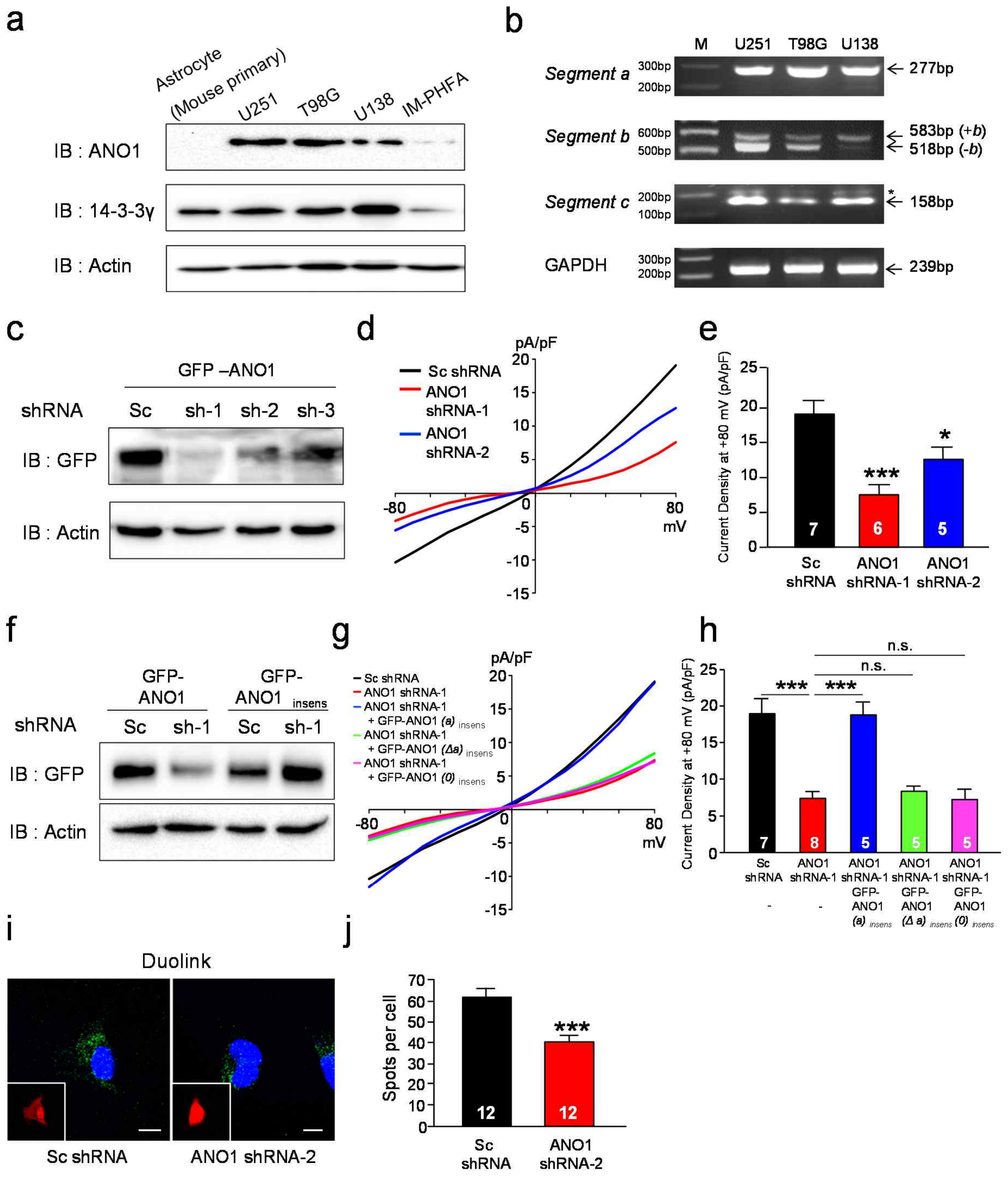

a
Astrocyte
(Mouse primary)
U251
T98G
U138
IM-PHFA
IB : ANO1
IB : 14-3-3γ
IB : Actin
b
M
U251
T98G
U138
Segment a
300bp
200bp
277bp
Segment b
600bp
500bp
583bp (+b)
518bp (-b)
Segment c
200bp
100bp
158bp
GAPDH
300bp
200bp
239bp
c
GFP –ANO1
shRNA
Sc
sh-1
sh-2
sh-3
IB : GFP
IB : Actin
d
pA/pF
Sc shRNA
ANO1 shRNA-1
ANO1 shRNA-2
mV
e
Current Density at +80 mV (pA/pF)
Sc shRNA
ANO1 shRNA-1
ANO1 shRNA-2
f
GFP-ANO1
GFP-ANO1 insens
shRNA
Sc
sh-1
IB : GFP
IB : Actin
g
Sc shRNA
ANO1 shRNA-1
ANO1 shRNA-1 + GFP-ANO1 (a) insens
ANO1 shRNA-1 + GFP-ANO1 (Δa) insens
ANO1 shRNA-1 + GFP-ANO1 (0) insens
pA/pF
mV
h
n.s.
Current Density at +80 mV (pA/pF)
Sc shRNA
ANO1 shRNA-1
GFP-ANO1 (a) insens
GFP-ANO1 (Δ a) insens
GFP-ANO1 (0) insens
i
Duolink
Sc shRNA
ANO1 shRNA-2
j
Spots per cell
Sc shRNA
ANO1 shRNA-2

Supplementary Figure 5. Endogenous ANO1 is expressed in glioblastoma cell lines. (a) ANO1 protein levels were determined in the cultured mouse astrocyte, human glioblastoma cell lines, U251, T98G and U138, and human immortalized primary human fetal astrocytes (IM-PHFA). (b) Identification of alternative splicing variants of ANO1 in glioblastoma cell lines. RT-PCR data showed that *segment a*-containing ANO1 is expressed in U251, T98G and U138 cells. The black arrows indicated the size of PCR products, and the inclusion (+) or exclusion forms (-) are indicated. All PCR products were confirmed by sequencing. M, 100-bp DNA size marker. An asterisk marks a non-specific band. (c) The efficiency of ANO1 shRNA constructs was tested by Western blotting in HEK293T cells. (d) Representative traces of whole-cell currents in U251 cells transfected with Sc shRNA or ANO1 shRNAs. (e) Summary bar graph showing normalized current density as in (d) at +80 mV. Data are expressed as mean±SEM; ***P<0.001, *P<0.05. (f) Western blot showing ANO1-insensitive form in HEK293T cells transfected with ANO1 shRNA-1. (g) Representative traces of whole-cell currents in U251 cells transfected with Sc shRNA, ANO1 shRNA and/or GFP-ANO1 $(a)_{insens}$, GFP-ANO1 $(\Delta a)_{insens}$ and GFP-ANO1. $(0)_{insens}$. (h) Summary bar graph showing normalized current density as in (g) at +80 mV. Data are expressed as mean±SEM; n.s.=not significant, ***P<0.001. (i) Representative images of Duolink PLA assay in U251 cells transfected with Sc shRNA and ANO1 shRNA-2. Scale bar, 10 µm. (j) PLA signals were counted with Duolink Image tool software, and the average number of spots per cell is presented. Data are expressed as mean±SEM; ***P<0.001.

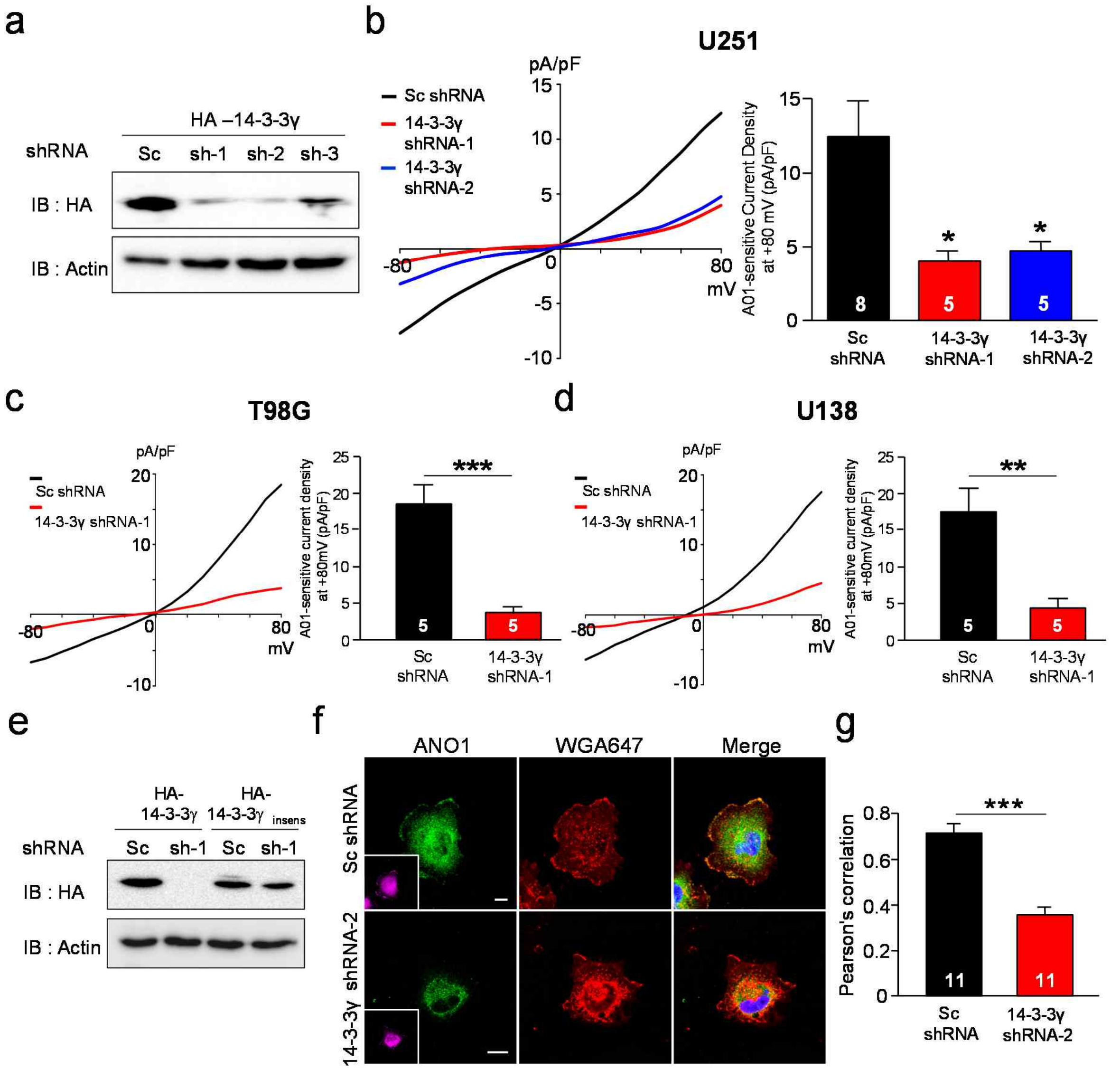

Supplementary Figure 6. Validation of 14-3-3γ shRNA constructs. (a) The efficiency of 14-3-3γ shRNA constructs was tested by Western blotting in HEK293T cells. (b) Representative traces of whole-cell currents in U251 cells transfected with Sc shRNA or 14-3-3γ shRNAs γ (left panel). Summary bar graph showing normalized current density as in left panel at +80 mV (right panel). Data are expressed as mean±SEM; *P<0.05. (c, d) Representative traces of A01-sensitive currents in T98G (c) and U138 (d) cells transfected with Sc shRNA, 14-3-3γ shRNA-1, and/or a GFP-14-3-3γ-insensitive form of 14-3-3γ (left panel). Summary bar graphs showed normalized A01-sensitive current density as in left panel at +80 mV (right panel). Data are expressed as mean±SEM; ***P<0.001, **P<0.01. (e) Western blot showing 14-3-3γ-insensitive form in HEK293T cells transfected with 14-3-3γ shRNA-1. (f) Representative immunocytochemical images of U251 cells transfected with Sc shRNA or 14-3-3γ shRNA-2. Scale bar, 10 μm. (g) Pearson's correlation coefficients denoting covariance of fluorescence signals were calculated using Nikon software as in (f). Data are expressed as mean±SEM; ***P<0.001.

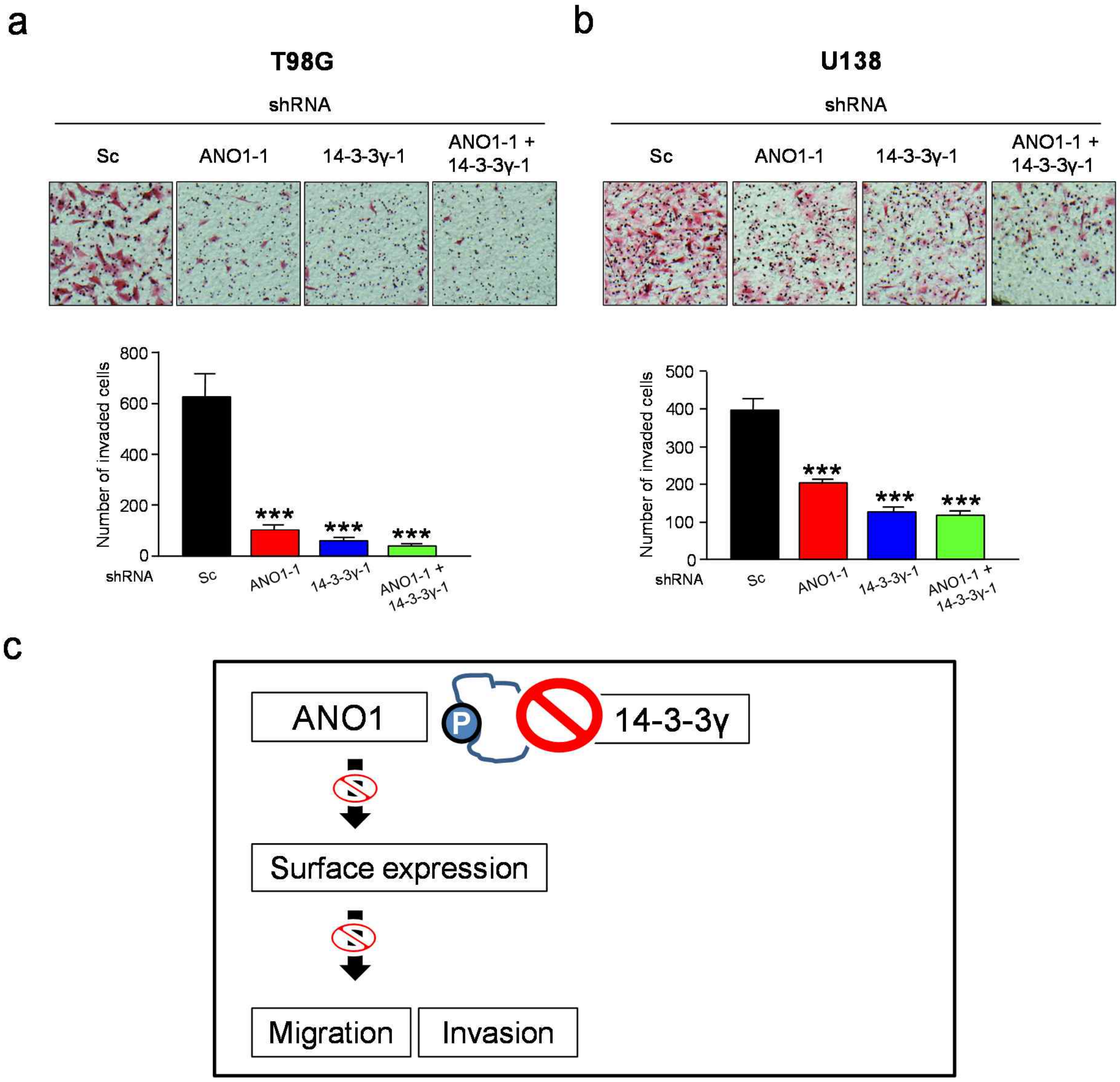

Supplementary Figure 7. Silencing of 14-3-3γ inhibits invasion of T98G and U138 cells. (a, b) Representative images (upper panel) of collagen-coated transwell invasion assay in T98G (a) and U138 (b) cells infected with Sc shRNA, 14-3-3γ shRNA-1, and/ or ANO1 shRNA-1. Normalized bar graphs (lower panel) showing data obtained from three independent experiments as in (upper panel). Data are expressed as mean±SEM; ***P<0.001. (c) A schematic diagram shows the inhibitory effects of 14-3-3γ silencing on migration and invasion via interfering the surface expression of ANO1.

Supplementary Methods

Cell culture

Primary mouse astrocyte were isolated from the cortex of P1 to P3 mice and maintained in DMEM (Gibco) as described[1]. IM-PHFA (immortalized primary human fetal astrocytes) cells were cultured as described[2].

Cell surface expression assay

For surface expression analysis, an HA tag (YPYDVPDYA) was inserted at the extracellular first loop of ANO1. HA-inserted ANO1 (ANO1[HA]) and FLAG-14-3-3γ were transfected into HEK293T cells. After 24 h, transfected cells were incubated in PBS containing 5% bovine serum albumin for 30 min at 4°C to block non-specific binding of the primary antibody. Cells were then incubated with anti-HA antibody (3F10, Roche) in PBS containing 2% bovine serum albumin for 1 h at 4°C, washed three times with ice-cold PBS, and incubated with a HRP-conjugated goat anti-rat antibody (Jackson Lab) in PBS containing 2% fetal bovine serum for 1 h at 4°C. Cells were gently washed three times with ice-cold PBS and lysed for 30 min on ice in lysis buffer. Samples were quantified in a luminometer using TECAN. The surface expression of each construct was analyzed in the same experiment, and at least three experiments were carried out.

References

1 Hwang, E. M. *et al.* A disulphide-linked heterodimer of TWIK-1 and TREK-1 mediates passive conductance in astrocytes. *Nature communications* **5**, 3227, doi:10.1038/ncomms4227 (2014).

2 Lee, S. G. *et al.* Astrocyte elevated gene-1 activates cell survival pathways through PI3K-Akt signaling. *Oncogene* **27**, 1114-1121, doi:10.1038/sj.onc.1210713 (2008).
